# The correlation of starch composition, physicochemical and structural properties of different sorghum grains

**DOI:** 10.3389/fpls.2025.1515022

**Published:** 2025-02-25

**Authors:** Kuangye Zhang, Fulai Ke, Hanling Zhou, Jiaxu Wang, Zhenbing Ma, Fei Zhang, Yanqiu Wang, Zhipeng Zhang, Feng Lu, Youhou Duan, Han Wu, Linlin Yang, Zidan Yang, Kai Zhu, Jianqiu Zou

**Affiliations:** ^1^ Sorghum Research Institute, Liaoning Academy of Agricultural Sciences, Shenyang, Liaoning, China; ^2^ Wuliangye Yibin Co., Ltd., Yibin, Sichuan, China; ^3^ Sichuan Province Engineering Technology Research Center of Liquor-Making Grains, Yinbin, Sichuan, China

**Keywords:** sorghum, starch, chain length distribution, particle size distribution, thermodynamic properties

## Abstract

The composition, structure, and physicochemical properties of starch in sorghum grains greatly influence the processing and quality of the final products. In this study, 19 sorghum lines were examined to analyze various starch-related characteristics. Correlation analysis of these key traits, revealed a significant correlation between amylose and amylopectin content. Amylopectin was identified as the primary component, averaging 80.75% of the starch content. The distribution of starch chain lengths, as well as the degrees of polymerization and branching, varied significantly among the sorghum lines, maintaining an equilibrium relationship between chain lengths. The size distribution of starch granules also varied among the lines, showing an overall positive correlation. Thermodynamic properties were positively correlated with each other, with correlation coefficients exceeding 0.614. Peak viscosity, trough viscosity, and final viscosity during the pasting process were highly correlated with the setback value, with correlation coefficients of -0.520, -0.651, and 0.618, respectively. 19 sorghum lines were classified into three categories: glutinous, japonica, semi-glutinous. Japonica sorghum exhibited superior thermal stability and viscoelasticity. This study elucidates the relationship between starch fractions, structure and physicochemical properties, providing a crucial theoretical foundation for optimizing sorghum processing for food and industrial applications.

## 1 Introduction

Sorghum, an ancient and vital crop, holds a pivotal position in global agriculture due to its resilience to drought, salinity and poor soil conditions. It is especially crucial in arid and semi-arid regions, where it serves as an indispensable cash crop. Beyond its role as a direct food source, sorghum is irreplaceable in industrial production, particularly within the starch industry (Zheng et al., 2023; Habyarimana et al., 2022; Silva et al., 2022).

Sorghum starch, a primary component of sorghum seeds, boasts excellent physicochemical properties. Its unique granular properties make it highly suitable for food processing and industrial applications, such as sauces, pastries and noodles due to its superior thickening, gelling and water retention capabilities (Kang et al., 2023; Xiao et al., 2022; McKinley et al., 2018). The chemical composition of sorghum starch includes starch, protein, fat, ash and moisture. Research indicates that starch content in different sorghum varieties ranges from 65 to 85 percent, protein content from 6 to 15 percent, and fat content from 0.5 to 2 percent (Kang et al., 2022). The main components of sorghum starch, amylose and amylopectin, and their proportion and distribution, determine the starch’s overall physicochemical properties and functional characteristics. Amylose is primarily connected by α-1,4 glycosidic bonds and has a long molecular chain, facilitating the formation a crystalline structure. In contrast, amylopectin contains branching points with α-1,6 glycosidic bonds, resulting in a complex molecular structure that affects its solubility and pasting properties in water differently from amylose (Mkandawire et al., 2013; Mutisya et al., 2003). Therefore, studying the component ratios and distribution of sorghum starch is crucial for a comprehensive understanding of its structure and properties. Additionally, sorghum starch is rich in various phytochemicals, including non-starch polysaccharides, proanthocyanidins, and tannins. These compounds not only impart unique nutritional value to sorghum starch but also open up possibilities for its application in medicine, cosmetics, and other fields (Ke et al., 2022).

The structural characteristics of sorghum starch, including granule morphology, size, crystallinity and lamellar structure, directly influence its solubility, pasting and aging properties in water (Yan et al., 2023). For instance, the size and shape of sorghum starch granules impact its dissolution rate and pasting behavior in food processing, while its crystallinity and lamellar structure determine its stability and resistance to aging in water (Li et al., 2021). Therefore, studying the structure of sorghum starch is essential not only for understanding the intrinsic mechanisms behind its physicochemical properties but also providing theoretical support to improve its processing performance and develop new products. Most sorghum starch granules are irregularly shaped with concave surfaces and range in size from 5 to 20 μm. Some granules exhibit a honeycomb-like surface structure, while a few are spherical with smooth surfaces (Vu et al., 2017). These granules can combine with water molecules to form a paste during heating. Different varieties of sorghum starch exhibit varying pasting characteristics. Some varieties of sorghum starch form pastes with high viscosity and good stability, while others can be hydrolyzed into sugar molecules through enzymolysis (Xiao et al., 2022; Zhong et al., 2021). Certain types of sorghum starch produce more sugar molecules during enzymatic hydrolysis, demonstrating a stronger saccharification ability is. In recent years, with advances in science and technology and improved living standards, research and application of sorghum starch have become increasingly in-depth. From the extraction process to modification technology studies, and from its use in food processing to its expansion into the medical and cosmetic fields, significant progress has been made (Castro-Campos et al., 2021). However, challenges remain, such as environmental pollution during production and the need to optimize and improve its physicochemical properties.

In practical applications, the most important properties of sorghum starch are its pasting, viscosity, gelation and retention. The pasting property refers to the ability of starch granules to combine with water molecules and form a paste when heated (Soe et al., 2022). Different varieties of sorghum starch exhibit varying pasting properties, influenced by the ratio of amylose to amylopectin, the structure of the starch granules, and environmental factors such as temperature and moisture content. Some varieties from high viscosity, well-stabilized pastes during heating, making them suitable for food or industrial products that require high viscosity (Griebel et al., 2021). In contrast, other varieties may have lower viscosity and stability, catering to different processing needs. Viscosity is a crucial physical property of sorghum starch during processing, reflecting the fluidity and consistency of the starch paste. This property significantly impacts the taste, texture and stability of the final product. Research indicates that viscosity parameters of sorghum starch, such as pasting temperature, peak viscosity, breakdown value, and final viscosity, differ notably among varieties (Griebel et al., 2019). These variations are primarily driven by genetic diversity, environmental factors, and the methods used for starch extraction (Sang et al., 2008; Salvati et al., 2024). Therefore, understanding sorghum starch properties requires considering these factors together to identify performance patterns and influencing factors in practical applications.

In recent years, the rapid advancement of the food industry, biotechnology and medicine, has spurred significant progress in research on the components, structure and properties of sorghum starch (Palavecino et al., 2020). Modern analytical techniques have enabled a deep understanding of the microstructure and molecular mechanisms of sorghum starch, uncovering its potential applications in food processing, drug delivery systems, and biomaterials. These findings provide crucial theoretical support for further research and development of sorghum starch and offer new insights and directions for the growth of related industries (Magallanes-Cruz et al., 2023).

In this study, 19 representative sorghum lines were analyzed to examine the starch fractions, structural characteristics, and physicochemical properties of their kernels. The aim was to explore the complex correlations between various indicators and establish a scientific model or indicator system. This system would provide a theoretical foundation for sorghum varietal improvement, food processing, and nutritional health research. The study also evaluated the applicability of different sorghum strains in food processing and their impact on product quality. The findings aim to enhance the basic scientific understanding of sorghum as a significant crop, and offer scientific and technical support for the comprehensive development and utilization of sorghum resources. Additionally, the results will aid in optimization and upgrading food processing technologies and promoting the sustainable development of agricultural production.

## 2 Materials and methods

### 2.1 Test materials and experimental design

Nineteen lines with varying starch content were selected from the F8 progeny of both 2381 and LNR-6 for testing. The total starch content of the 19 selected lines ranged from 48.00% to 72.77%, the amylose content ranged from 0 to 25.98% with a coefficient of variation of 57.49%, the amylopectin ratio ranged from 57.28% to 100%, and the coefficient of variation of the resistant starch content was 130.49%, which indicated that the distribution of various types of starch content of the 19 samples was wide, covering glutinous, japonica, highly resistant starch and other types of starch, which is a very good representation.

The experiment took place in 2022 and 2023 at the West Experimental Site of the Liaoning Academy of Agricultural Sciences (123.56°E, 41.82°N). A completely randomized block design was employed, featuring a 4-meter row length, 0.6 m row spacing, 0.2 m plant spacing, three row zones, three replications, and standard field management practices.

After harvesting, the seeds were dried, dehulled and sieved to eliminate impurities. The cleaned samples were equilibrated for one week in a constant temperature and humidity cabinet. Subsequently, the samples were ground, dispersed in a mortar, and passed through a 100-mesh sieve. The processed samples were then stored in a low humidity cabinet for future use.

### 2.2 Determination of starch content

#### 2.2.1 Determination of total starch content

Weigh an appropriate amount of sample (approximately 50mg dry, 100mg wet) into a 15mL centrifuge tube. Add 2ml of 80% ethanol, mix well, and leave at 70°C for 2h, vortex and mix well during the period, and cool to room temperature. Add 2mL deionized water, mix well, centrifuge at 12000rpm for 10min, discard supernatant. Add 4mL 80% ethanol, mix well, centrifuge at 12000rpm for 10min, discard the supernatant, repeat twice. Add 2mL 2M KOH, mix the tube upside down and shake on ice for 20min. add 8mL 1.2M sodium acetate buffer solution (pH=3.8) and vortex well. Add 0.1 mL of heat-resistant α-amylase and 0.1 mL of amyloglucosidase, vortex well, and incubate at 50°C for 30 min, during which time vortex well. For samples containing 1-10% starch, the volume was adjusted to 10 mL directly with distilled water; for samples containing 10-100% starch, 0.1 mL of the sample was diluted to 1 mL with distilled water and the reaction was carried out. Take 0.05mL of the above liquid into a new EP tube, add 1.5mL of GOPOD reagent, vortex and mix well, incubate at 50°C for 20min. take 0.05mL of glucose standard solution, add 1.5mL of GOPOD reagent, and react at 50°C for 20min. measure the absorbance at 510nm. The SC of the sample can be calculated according to the following formula:

SC<10%:


$$SC\left(\%\right)=\frac{\text{ΔA }\times \text{ F }\times 9.27}{\text{M}}$$

SC>10%:


$$SC\left(\%\right)=\frac{\text{ΔA }\times \text{ F }\times 90}{\text{M}}$$

ΔA : Sample OD - blank OD;

F: OD value per μ g of glucose;

M: Weight of the sample.

Amylose content (AC) was determined by the dual wavelength method with main and reference wavelengths (λ1 and λ2, respectively) of 620 nm and 479 nm for amylose content (Ke et al., 2022). The AC in the sample can be calculated according to the standard curve:


AC=C÷M×10

where C is the result calculated from the standard curve; M is the actual weighing mass (generally 10 mg); and 10 is the correction factor.

#### 2.2.2 Determination of resistant starch

Sorghum seed resistant starch extraction was referred to Palavecino et al. and Teixeira et al (Palavecino et al., 2020; Teixeira et al., 2016). The non-resistant starch was removed mainly using α-tryptic amylase and amyloglucosidase, the residue was dissolved and adjusted to neutrality, the starch was hydrolyzed to glucose using amyloglucosidase, stained and then its content was determined by colorimetric method (Palavecino et al., 2020; Teixeira et al., 2016).The quantitative analysis of the target was carried out using a Thermo Fisher (USA) multifunctional enzyme labeler, instrument model Multiskan GO. The optimum UV absorption wavelength (510 nm) was confirmed by scanning the reaction solution photometry at full wavelength. The enzyme labelling plates were made of Corning (USA), which requires less than 0.02 difference between wells at the target wavelength.

#### 2.2.3 Amylopectin content, the ratio of amylopectin to amylose and the amylopectin ratio


ATC=SC−AC

ATA=ATC÷AC

ATR=ATR÷SC×100%

### 2.3 Determination of chain length distribution and branching degree of starch

Starch chain length distribution index, mainly using ion chromatography to separate different chain lengths of glucose chains after enzymatic digestion. The chain length distribution of branched starch refers to the hydrolysis of the 1,6-glycosidic bond of branched starch by isoamylase, and the final hydrolysis of branched starch into glucose chains of different lengths, and then analyze the content of glucose chains with different degrees of polymerization by high-performance anion-exchange chromatography with pulsed amperometric detection (HPAEC-PAD) method. For chromatographic analysis we used a Thermo ICS5000 ion chromatography system with a Dionex™ CarboPac™ PA200 (250*4.0 mm, 10 um), with an injection volume of 5 uL. The mobile phases included an A phase: 0.2 M NaOH; and a B phase: 0.2M NaOH/0.2 M NaAC. We set the column temperature to 30 °C. Detection was performed using an electrochemical detector. Flow rate: 0.4 ml/min. Elution gradient was: 0min A/B (90:10 V/V), 10 min A/B (90:10 V/V), 30 min A/B (40:60 V/V), 50 min A/B (40:60 V/V), 50.1 min A/B (90:10 V/V), 60 min A/B (90:10 V/V). The peak areas of samples DP6-DP76 were summed to obtain the total area. The Relative Area for each chain length was calculated as follows: Relative Area= Area/Total Area*100%. The distribution of chain lengths was characterized using Polymerization Metricity (PMT) (Kang et al., 2022).

### 2.4 Characteristics of starch particle size distribution

In this experiment, the starch particle size distribution in the samples was examined by laser particle sizer analysis method, and the specific analysis conditions and analysis methods are as follows: The spectroscopy system was carried out using a Malvern laser particle sizer Matersizer 3000 (Malvern Instruments Ltd, Worcestershire, UK) for wet detection, equipped with a Hydro LV wet disperser with an irregular structural model, ultrapure water as solvent, and a shading coefficient of 1.3330 and a starch shading coefficient of 1.50; the shading range was 12-17%, and the instrument was cleaned and calibrated before each sample determination (Trappey et al., 2015). The response particle size distribution characteristic indexes are represented by the indices Dx(10), Dx(50) and Dx(90) for quantity, volume and surface area. These are recorded as Num (10), Num(50), Num(90), Vol(10), Vol(50), Vol(90), Sur(10), Sur(50), and Sur(90), respectively.

### 2.5 Determination of thermodynamic properties of starch

The sample was equilibrated for 24 hours at room temperature. A differential scanner calorimeter (DSC, Q2000, TA Instruments, USA) was used to scan the calorimetric changes from 30°C to 95°C at a rate of 10°C per minute. The data were analyzed using Universal Analysis software. The onset temperature (TO), peak temperature (TP), termination temperature (TC) and enthalpy of pasting (ΔH) were calculated to characterize the phase transformation process of the samples (Oladele et al., 2019).

### 2.6 Determination of starch pasting properties

We removed the samples from moisture equilibrium, ground and dispersed them in a mortar, and passed them through a 100-mesh sieve. The sieved samples were stored in a low-humidity cabinet. An appropriate amount of the moisture-equilibrated sample was weighed and added to a moisture meter to detect and record the moisture content. The sample viscosity was measured based on its moisture content. After this, we weighed the sample precisely, ensuring an error lower than 0.05 g. The sample was placed into aluminum foil and 25 g of sterile water were added and mixed thoroughly. Then, we used the RVA Super 4 Rapid Viscosity Analyzer to quickly measure the sample’s viscosity (Oladele et al., 2019). The following parameters were measured: Peak Viscosity (PV), Trough Viscosity (TV), Breakdown Value (BV), Final Viscosity (FV), Setback Value (SV), Peak Time (Ptime), and Peak Temperature (PaTem).

### 2.7 Data-processing methods

Basic phenotyping, correlation analysis, cluster analysis, and analysis of variance (ANOVA) were performed using Microsoft Excel and SPSS 24.0 software.

## 3 Results

### 3.1 Sorghum grain starch content

**Figure 1:**
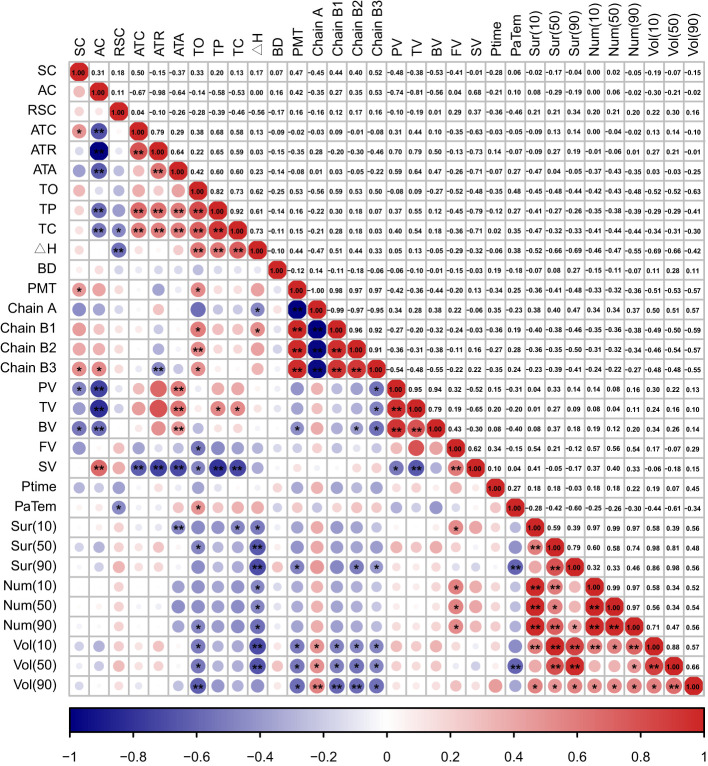
Heat map for correlation analysis of starch traits in sorghum grain. “*” indicates p<0.05; “**” indicates p<0.01.

**Figure 1** presents the results for total amylose content, branched chain, resistant amylose content, branched chain amylose percentage, and the straight-to-branched ratio for the 19 sorghum lines. The average amylose content across these varieties was 59.55% with a coefficient of variation (CV) of 9.47%. Line 4 had the lowest amylose content of 48.00%, while line 17 had the highest at 72.77% (**Table 1**). The mean branched amylose content was 11.55%, with a CV of 57.49%. Notably, line 11 had zero amylose content, possibly due to a unique starch synthesis mechanism or a gene mutation, whereas line 14 exhibited the highest branched amylose content at 25.98%, indicating its superior amylose accumulation (**Table 1**). For resistant starch, the average content among the 19 lines was 8.07%, with substantial variation indicated by a CV of 127.01%. Line 18 had the lowest resistant starch content at 0.18%, while line 9 had the highest at 34.55% (**Table 1**). Regarding branched starch, the mean content and proportion were 48.00% and 80.75%, respectively, with CVs of 15.16% and 13.49%. Line 14, had the lowest values for both branched starch content and proportion at 34.84% and 57.28%, respectively, while line 11 had the highest branched amylose content and ratio, at 64.23% and 100% (**Table 1**). The mean branched ratio for the 19 sorghum lines was 7.79, with a coefficient of variation (CV) of 145.14%. Line 11 had the smallest straight-to-branched ratio at 0%, while line 18 had the largest at 40.39% (**Table 1**).

**Table 1:** Parametric analysis of starch fractions.

Items	SC (%)	AC (%)	RSC (%)	ATC (%)	ATR (%)	ATA (%)
AVG	59.55	11.55	8.07	48.00	80.75	7.79
SD	5.64	6.64	10.54	7.27	10.90	11.31
CV	9.48	57.49	130.49	15.16	13.49	145.14
min	48.00	0	17.92	34.84	57.28	0
max	72.77	25.98	34.55	64.23	100.00	40.39

Correlation analysis revealed several highly significant negative correlations among the traits: AC and ATC (r=-0.675, p=0.002), AC and ATR (r=-0.984, p<0.001), and AC and ATA (r=-0.638, p=0.003). Additionally, there were two groups of highly significant positive correlations: ATR and ATA (r=0.639, p=0.003), and ATR and ATC (r=0.785, p<0.001). Another significant positive correlations was found between ATC and SC(r=0.496, p=0.031) (**Figure 1**; **Supplementary Table S1**). These results indicate competition between straight chain and branched chain starch during sorghum starch synthesis, reflecting the genotypic preference for synthesizing these starch types. Branched chain starch, being the main component of sorghum starch, contributes directly to the increase in total starch content.

### 3.2 Analysis of chain length distribution and branching degree of seed starch

The PMT for each sorghum line is presented in **Table 2**. The mean polymerization value across the 19 lines was 33.94, with a significant variation in starch chain length indicated by a CV of 8.18%. Line 1 exhibited the smallest average polymerization of 25.86, while line 17 had the largest at 37.51.The distribution of the starch chain lengths of the 19 lines was analyzed and the results were shown in **Supplementary Figure S1**. Analysis of the starch chain length distribution showed varied patterns across the lines. Five lines (1, 2, 3, 4 and 5), displayed no peak distribution. Of these, line 1 had the largest relative peak area ratio near DP6, while lines 2, 3, 4 and 5 had their largest relative peak area ratios near DP7. The remaining 13 lines exhibited a similar distribution with a single peak. Specifically, lines 6, 7, 8, 10, 12, 13, 14, 15, 16 and 19 had their highest peaks at DP11, lines 9, 17 and 18 peaked at DP12, and line 11 peaked at DP10. These results indicate a specific interval preference in the amylose chain length distribution among different lines.

**Table 2:** Parametric analysis of chain length distribution characteristics.

Items	Chain A (%)	Chain B1 (%)	Chain B2 (%)	ChainB3 (%)	PMT	BD (%)
Avg	35.02	48.61	10.54	5.83	33.94	5.75
SD	8.03	5.51	1.30	1.33	2.78	0.97
CV	22.92	11.33	12.30	22.77	8.18	16.90
Min	24.66	33.95	6.44	2.03	25.86	4.11
Max	57.59	55.78	11.91	7.66	37.51	7.46

The branched starch chain length distribution was classified into four groups based on the degree of polymerization (DP): A chain (DP6-DP12), B1 chain (DP13-DP24), B2 chain (DP25-DP38) and B3 chain (DP>38). The branched starch chain lengths predominantly fell within the A chain and B1 chains. Lines 1, 2 and 3 had a higher percentage of A-chain amylopectin, with values of 57.59%, 46.03% and 44.15%, respectively, followed by B1 chain percentages of 33.95%, 40.91% and 42.19%, respectively. The other 16 lines exhibited the highest content in the B1 chain, with a mean value of 50.41%, followed by the A chain. This indicates that A and B1 chains are the major components of amylopectin in sorghum and that the composition of these chains varies among different lines (**Table 2**).

The BD is a parameter that describes the number of branching chains in a starch molecule, influencing its solubility, pasting temperature, and gelatinization characteristics. The seed starch branching degree results for each line are shown in **Table 2**. the average branching degree across the 19 lines was 5.75%, with a CV of 16.93%, and a range from 4.11% to 7.46%. The three lines with the smallest starch branching degrees were line 2, line 10, and line 19. Conversely, the three lines with the largest starch branching degrees were line 12, line 13, and line 1. A high branching degree can affect starch properties such as solubility and pasting temperature, thereby impacting the processing quality and nutritional value of sorghum.

Correlation analysis of chain length-related traits revealed that, except for BD, which showed no significant correlation with the other five traits, there were highly significant correlations among the remaining five traits. Specifically, there were six positive correlations and four negative correlations (**Figure 1**; **Supplementary Table S1**).The correlation between PMT and the ratios of B1, B2, and B3 chains were highly significant and positive, with correlation coefficients of 0.978, 0.989 and 0.985, respectively. In contrast, the correlation between PMT and the A-chain ratio was highly significant and negative (r=-0.994, p<0.001). Additionally, there were highly significant positive correlations between the A-chain ratio and B-chain ratio, as well as among the B-chain ratios. These findings suggest that an increase in PMT promotes the formation of longer starch chains, indicating an equilibrium between short and long chains. Furthermore, the ratios of B chains of different lengths in the starch molecule vary synergistically.

### 3.3 Starch particle size distribution characteristics

The size distribution characteristics of starch grains in sorghum kernels are crucial indicators for assessing their quality and utilization value. The distribution trends of starch grains among the test varieties were similar, showing a single peak in terms of surface area, number and volume (**Supplementary Figures S2A–C**). For surface area, the peaks among the 19 lines ranged from 9.85 µm to 12.73 µm, indicating a concentration trend in starch grain surface area across different lines. Specifically, lines 1, 2, 3, 7 and 18 peaked at 12.73 µm; lines 4, 5, 6, 8, 9, 10, 11, 12, 13, 14, 15, 17 and 19 peaked at 11.20 µm; and line 16 peaked at 9.86 µm, reflecting the variations in starch granule surface area among the lines. The Dx(10) index, representing the size below which 10% of the grains fall, ranged from 3.37 µm to 8.11 µm, with a mean of 6.84 µm and a coefficient of variation (CV) of 14.96%. The Dx(50) index, representing the median grain size, ranged from 10.72 µm to 14.73 µm, with a mean of 12.97 µm and a CV of 6.48%. The Dx(90) index, indicating the size below which 90% of the grains fall, ranged from 23.07 µm to 52.30 µm, with an average of 30.23 µm and a CV of 23.75% (**Table 3**).

**Table 3:** Parametric analysis of particle size distribution characteristics.

Items	Sur (10)	Sur (50)	Sur (90)	Num (10)	Num (50)	Num (90)	Vol (10)	Vol (50)	Vol (90)
Avg (μm)	6.84	12.97	30.23	5.18	7.97	14.73	9.20	20.23	96.02
SD (μm)	1.05	0.86	7.38	1.15	1.71	1.63	0.70	3.38	20.59
CV (%)	15.37	6.66	24.40	22.23	21.50	11.06	7.61	16.73	21.44
Min (μm)	3.37	10.72	23.07	1.92	2.60	10.29	7.53	16.25	55.75
Max (μm)	8.11	14.73	52.30	6.24	9.59	16.66	10.87	28.60	140.30

Regarding the quantitative distribution of the 19 sorghum lines, most lines (17 out of 19) had peaks within the 6.72 µm to 8.68 µm range, with line 3 peaking at 8.68 µm and lines 2, 4, 15 and 19 peaking at 7.64 µm. However, lines 16 and 18 had peaks at 2.13 µm, indicating a different distribution trend. The Dx(10) index, which indicates the size below which 10% of the grains fall, ranged from 1.92 µm to 6.24 µm. Line 18 had the smallest Dx(10) index, while line 3 had the largest. The mean Dx(10) index across the 19 lines was 5.18 µm, with a coefficient of variation (CV) of 21.64%. The Dx(50) index, representing the median size, ranged from 2.60 µm to 9.59 µm. Line 18 again had the smallest Dx(50) index, and line 3 had the largest. The mean Dx(50) index was 7.97 µm with a CV of 20.93%.The Dx(90) index, indicating the size below which 90% of the grains fall, ranged from 10.29 µm to 16.66 µm. Line 18 had the smallest Dx(90) index, while line 3 had the largest. The mean Dx(90) index was 14.73 µm with a CV of 10.77% (**Table 3**).

In terms of volume distribution, six out of the 19 sorghum lines exhibited peaks at 16.43 µm, specifically lines 1, 3, 7, 13, 15 and 18. The remaining 13 lines had peaks at 14.46 µm. The Dx(10) index, representing the size below which 10% of the grins fall, ranged from 7.53 µm to 10.87 µm. Line 16 had the smallest Dx(10) index, while line 3 had the largest. The average Dx(10) index across the 19 lines was 9.20 µm, with a coefficient of variation (CV) of 7.41%.The Dx(50) index, indicating the median size, ranged from 16.25 µm to 28.60 µm. Again, line 16 had the smallest Dx(50) index, and line 3 had the largest. The mean Dx(50) index across the 19 lines was 20.23 µm, with a CV of 16.28%.The Dx(90) index, indicating the size below which 90% of the grains fall, ranged from 55.75 µm to 140.30 µm. Line 18 had the smallest Dx(90) index, while line 8 had the largest. The mean Dx(90) index across the 19 lines was 96.02 µm, with a CV of 20.87% (**Table 3**).

The Dx(10), Dx(50) and Dx(90) indices representing surface area, number and volume of starch granules were used to analyze the distribution characteristics of starch granule size through correlation analysis. All correlations between the distribution characteristics of starch particle size showed positive associations, with most correlations being significant or highly significant. The exceptions were the correlations between Vol(50) and Sur(10), Num(50), and Sur(90) and Num(50), and Num(90), which were not significant(**Figure 1**; **Supplementary Table S1**). These findings indicate that the Dx (10), Dx (50) and Dx (90) indices of surface area, number and volume of starch grains are generally correlated with each other. An increase in the number of starch grains within a specific size range tends to correspond with increases in their surface area and volume. However, significant variations were observed in the formation and distribution of starch grains across specific size ranges among different lines, suggesting that the correlation between volume, surface area and number of starch grains within these specific size ranges was relatively weak.

### 3.4 Analysis of thermodynamic properties of starch

Thermodynamic characterization of seed starch from 19 sorghum lines was conducted and the results for TO, TP, TC and ΔH are presented in **Table 4** and **Supplementary Figure S3**. The TO varied among the 19 lines, ranging from 65.17°C to 70.62°C, with a mean of 69.53°C. The three lines with the lowest TO were line 1 (65.17°C), line 4 (65.37°C) and line 8 (65.77°C). These temperatures are advantageous for applications requiring lower processing temperatures. Conversely, the three lines with the highest TO were line 19 (70.62°C), line 17 (70.28°C), and line 18 (69.54°C), suitable for applications needing higher temperature processing. The average TP across the lines was 72.11°C, ranging from 70.08°C to 75.29°C. The three lines with the lowest TP were line 4 (70.08°C), line 8 (70.45°C) and line 7 (70.49°C), indicating quicker attainment of maximum viscosity during pasting. In contrast, the three lines with the highest TP were line 19 (75.29°C), line 11 (74.63°C) and line 18 (74.55°C), likely due to their more stable starch crystal structure. The average TC among the 19 sorghum lines was 77.89°C, with temperatures ranging from 75.10°C to 81.86°C. The three lines with the lowest TC were line 4 (75.10°C), line 7 (75.32°C) and line 3 (75.32°C), which can enhance processing efficiency. Conversely, the three lines with the highest TC were line 19 (80.69 °C), line 18 (81.56 °C) and line 11 (81.86 °C). ΔH generally denotes the energy required to disrupt starch crystal regions during thermodynamic changes. The average ΔH of the 19 lines in this study was 10.56 J/g, ranging from 7.60 J/g to 12.19 J/g, with a coefficient of variation of 12.47%. The three lines with the lowest paste ΔH line 3 (7.60 J/g), line 7 (8.43 J/g), and line 1 (9.37 J/g). Conversely, the three lines with the highest ΔH were line 17 (12.19 J/g), line 6 (12.13 J/g) and line 18 (12.11 J/g).

**Table 4:** Parametric analysis of thermodynamic properties.

Items	TO (°C)	TP (°C)	TC (°C)	ΔH (J/g)
Avg	67.36	72.11	77.89	10.56
SD	1.65	1.47	2.08	1.32
CV (%)	2.45	2.18	2.66	12.47
Min	65.17	70.08	75.10	7.60
Max	70.62	75.29	81.86	12.19

There were highly significant positive correlations between the thermodynamic correlation indices. The correlation coefficients between the ΔH and the TO, TP,TC were 0.617, 0.614 and 0.732, respectively. Additionally, the correlation coefficients between the TC and the TO, TP were 0.728 and 0.919, respectively, while the correlation coefficient between the TO and the TP was 0.822 (**Figure 1**; **Supplementary Table S1**). As temperature gradually increases, the energy required for starch molecules to transition from an ordered to a disordered state also increases. This energy rise is closely related to the disruption of the starch crystal structure and the stretching of molecular chains, which are key features of the starch pasting process. The synergistic changes among the three temperature points collectively form the temperature curve of the starch pasting process.

### 3.5 Analysis of starch pasting properties

The starch pasting characteristics of sorghum grain are presented in **Table 5** and **Supplementary Figure S4**. The average peak viscosity for the 19 varieties was 1333.42 cp, with a coefficient of variation of 21.79%. The PV ranged from 823 cp (line 17) to 1891 cp (line 19). The average TV was 750.89 cp, with a coefficient of variation of 22.2%, and ranged from 487 cp (strain 17) to 1157 cp (strain 19). The mean BV for the 19 strains was 582.53 cp, with a coefficient of variation of 24.11%. The BV varied from a minimum of 313 cp (strain 16) to a maximum of 847 cp (strain 1). The mean value of FV was 1286.37 cp with a coefficient of variation of 12.51%. Among the 19 lines, the lowest FV was 922 cp (line 17), and the highest was 1555 cp (line 4). The average SV was 535.47cp, with a coefficient of variation of 38.85%. The SV ranged from 119 cp (line 18) to 866 cp (line 4). The mean Ptime for the 19 lines was 4.32 minutes, with a relatively small variation among varieties. The coefficient of variation was only 2.85%, and the Ptime ranged from 4.07 minutes to 4.53 minutes, indicating that the time to reach peak viscosity during the pasting process was relatively consistent across varieties. The PaTem of all varieties ranged from 75.2°C to 77.65°C, showing minimal variation among them and a coefficient of variation of only 1.19%.The average PaTem for the 19 varieties was 76.77°C, indicating similar temperature conditions required during the pasting process across all varieties.

**Table 5:** Parametric analysis of pasting characteristics.

Items	PV (cp)	TV (cp)	BV (cp)	FV (cp)	SV (cp)	Ptime (min)	PaTem (°C)
Avg	1333.42	750.89	582.53	1286.37	535.47	4.32	76.77
SD	290.55	166.70	140.45	160.92	208.03	0.12	0.91
CV(%)	21.79	22.20	24.11	12.51	38.85	2.85	1.19
Min	823.00	487.00	313.00	922.00	119.00	4.07	75.20
Max	1891.00	1157.00	847.00	1555.00	866.00	4.53	77.65

Correlation analysis between the seven pasting properties revealed a highly significant positive correlation between PV, TV, and BV, with correlation coefficients of 0.955, 0.935 and 0.788, respectively. Additionally, there was a negative correlation between SV and PV,TV, with correlation coefficients of -0.520 and -0.651, respectively. A highly significant positive correlation was found between SV and FV (**Figure 1**; **Supplementary Table S1**). The PV, PV, and BV are closely related in reflecting the starch pasting characteristics. The regrowth phenomenon of starch paste during the cooling process exhibits an antagonistic relationship with peak viscosity and trough viscosity during the heating process. A larger the SV indicates a more significant regrowth phenomenon during cooling, where starch molecules rearranged to form an ordered structure, leading to an increase in FV.

### 3.6 Correlation of starch fractions with structure and properties

To investigate the relationship between starch components, structure and properties, a correlation analysis was performed. The results indicated a few significant correlations between components and structure. Specifically, there were significant positive correlations between SC and PMT, as well as between B3 chains and AC (**Figure 1**; **Supplementary Table S1**). This suggests that longer starch chains are more likely to form a stable structure, enhancing the structural stability of starch granules and thereby improving SC. The linear structure of amylose molecules facilitates the addition of glucose units during synthesis, leading to the formation of longer molecular chains. Conversely, significant negative correlations were found between ATR and B3 chains, and between Sur(10) and ATA (**Figure 1**; **Supplementary Table S1**). The complex branching structure of amylopectin molecules restricts the elongation of starch chains and inhibits the formation of ultra-long-chain starch, thereby reducing the proportion of such starch. Although the starch structure is less influenced by its components, the content of certain specific components can still significantly impact its structural properties.

The results of the correlation analysis between starch fractions and indicators of thermodynamic properties and pasting characteristics revealed the following: SC: there was a significant negative correlation with PV and BV, but no significant correlation with the other indicators. AC: there was a significant or highly significant negative correlation with PV, TV, BV, TP, and TC. There was a highly significant positive correlation with SV. RSC: there were significant negative correlations with PaTem, TC, and ΔH. ATC: there were highly significant positive correlations with TP and TC, and a highly significant negative correlation with SV. ATR and ATA: Both indexes showed significant or highly significant positive correlations with PV, TV, BV, TP, and TC, and a highly significant negative correlation with SV (**Figure 1**; **Supplementary Table S1**). The results indicate that starch components significantly influence its thermodynamic properties and pasting characteristics. AC, in particular, has a broad and substantial impact on the pasting behavior of starch. An increase in AC decreases the viscosity of starch paste, affects the temperature range of pasting, and exacerbates the regrowth phenomenon the cooling. The rise in RSC levels might have altered the structure or composition of starch granules, influencing the temperature range of starch pasting and the ΔH during this process. Branched starch significantly contributes to enhancing the viscosity and stability of starch pastes, while also reducing the likelihood of retrogradation. An increase in branched starch content could strengthen the interactions between starch granules, forming a denser gel network and thereby improving the viscosity and stability of the starch paste.

### 3.7 Starch structure-property correlation analysis

Both chain length distribution and particle size distribution of starch are crucial factors influencing its properties and applications. Correlation analysis between these structural indexes revealed that the A chain has a significant or highly significant positive correlation with the four chain length distribution indexes: Vol(90), Vol(50), Vol(10) and Sur(90). Conversely, the B1, B2, and B3 chains and PMT showed a significant or highly significant negative correlation with these four particle size distribution indexes (**Figure 1**; **Supplementary Table S1**).

Correlation analysis of the thermodynamic properties and pasting characteristics of starch revealed a significant negative correlation between TO and FV and SV (**Figure 1**; **Supplementary Table S1**). When TO is lower, starch granules begin to paste at lower temperatures, allowing more starch molecules to participate in the pasting process. This results in the formation of a more compact network structure, reflected in the increase of FV. Additionally, the pasting process may be more uniform at lower temperatures, reducing the phenomenon of retrogradation, and thus decreasing SV. Furthermore, there is a significant positive correlation between TO and PaTem. This relationship likely arises from the physical characteristics of the pasting process of starch granules, where the thermal stability of starch influences the temperature at which pasting begins. The physical properties of the pasting process are influenced by the starting temperature. Higher starting temperatures require starch granules to reach a higher temperature to achieve peak pasting state. A significant positive correlation was observed between TP, TC and TV (**Figure 1**; **Supplementary Table S1**). Higher TP and TC indicate that starch granules need to reach a higher temperature to paste fully, leading to more thorough unfolding and mixing of starch molecules during pasting, which results in an increase in TV. Additionally, there was a highly significant negative correlation between TP, TC and SV. Higher TP and TC may enhance intermolecular cross-linking, making the paste structure more stable and less prone to retrogradation, thereby reducing the setback value. There was no significant correlation between ΔH and the pasting characteristics indicators. The ΔH during pasting mainly reflects the thermodynamic disorder of starch, which may not directly correlate with the pasting characteristics. Similarly, no significant correlations were found between PV, BV, Ptime, and the thermodynamic indicators.

To better understand the basis of starch properties, further correlation analysis between starch structural properties and physicochemical properties was conducted. The results showed significant correlations between the thermodynamic indexes (TO and ΔH) and the structural properties of starch. TO had significant or highly significant negative correlations with six indexes: A-chain, Vol(90), Vol(50), Vol(10), Num(90), and Sur(50). There was also a highly significant positive correlation between TO and the B1-chain (**Figure 1**; **Supplementary Table S1**). ΔH was mainly affected by the size distribution indexes. There was a significant or highly significant negative correlation between ΔH and all size distribution indexes except for Vol (90). Significant negative correlations were observed between ΔH and A chains, while significant positive correlations were seen with B1-chains. This suggests that the ΔH is influenced by the structural arrangement and size distribution of the starch granules, with different chain lengths playing distinct roles in the energy dynamics of the pasting process. There is a significant negative correlation between the TC and Sur (10). There is also a significant negative correlation between the pasting characteristics indexes PV, TV, and BV and the B3 chain. Additionally, BV and the B2 chain show a significant negative correlation. The B3 chain may influence hydration, particle swelling, and viscosity formation during the pasting process due to its specific chain length and structure. The B2 chain may affect the maintenance and stabilization of the particle structure during pasting. Significant positive correlations were found between FV and Num (90), Num (50), Num (10), and Sur (10). A greater number of particles and a larger specific surface area provide more contact points and interactions, thus increasing FV. Highly significant negative correlations were observed between PaTem and Vol (50), Sur (90).

**Figure 2:**
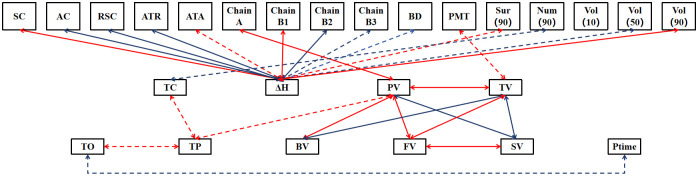
The path analyses of starch properties with components and structure. Only significant or highly significant associations are demonstrated in the figure, with red and blue arrows representing positive and negative associations, respectively, the solid and dashed lines representing significance at the 0.01 level and 0.05 level, respectively.

The results of path analyses of starch properties with components and structure showed that components and structural indicators were more likely to influence ΔH, including five significant or highly significant positive associations for SC, ATA, Chain B1, Sur(90), Vol(90) and seven significant or highly significant negative associations for AC, RSC, ATR, Chain B2, Chain B3, BD, Vol(50). However, the component and structural indicators have less influence on the other characteristic indicators, with only significant or highly significant associations between Num(90) and TC, Chain A and PV, PMT and TV. There are also some significant or highly significant associations between the 11 characteristic indicators (except PaTem), such as significant positive associations between TO and TP, significant negative associations between TO and Ptime; and a highly significant positive association between FV and PV, TV, SV. The components and structure of starch have significant and diverse effects on its thermodynamic properties (especially ΔH), and the complex and tight network of connections among the starch property indices suggests that they interact and influence each other. Therefore, in order to deeply understand and regulate the properties of starch, it is necessary to comprehensively consider the intrinsic connection between its components, structure and property indexes, with a view to achieving more precise and effective control (**Figure 2**).

### 3.8 Analysis of starch properties of different types of sorghum

**Figure 3:**
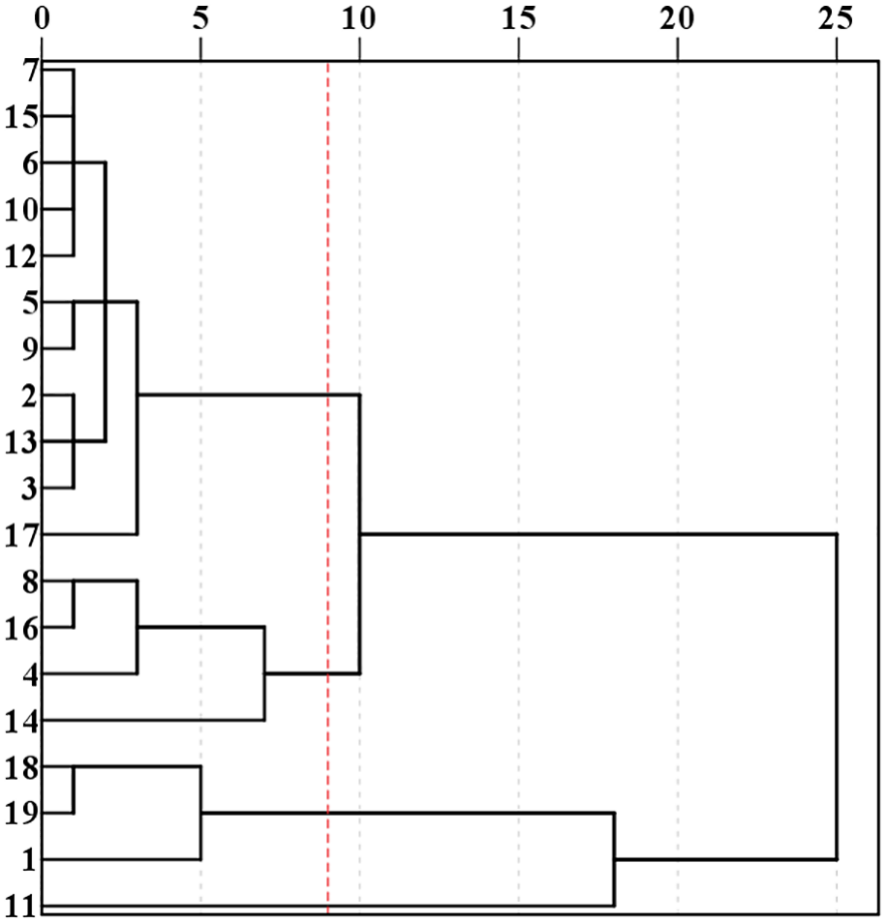
Results of systematic clustering for different sorghum lines.

**Figure 4:**
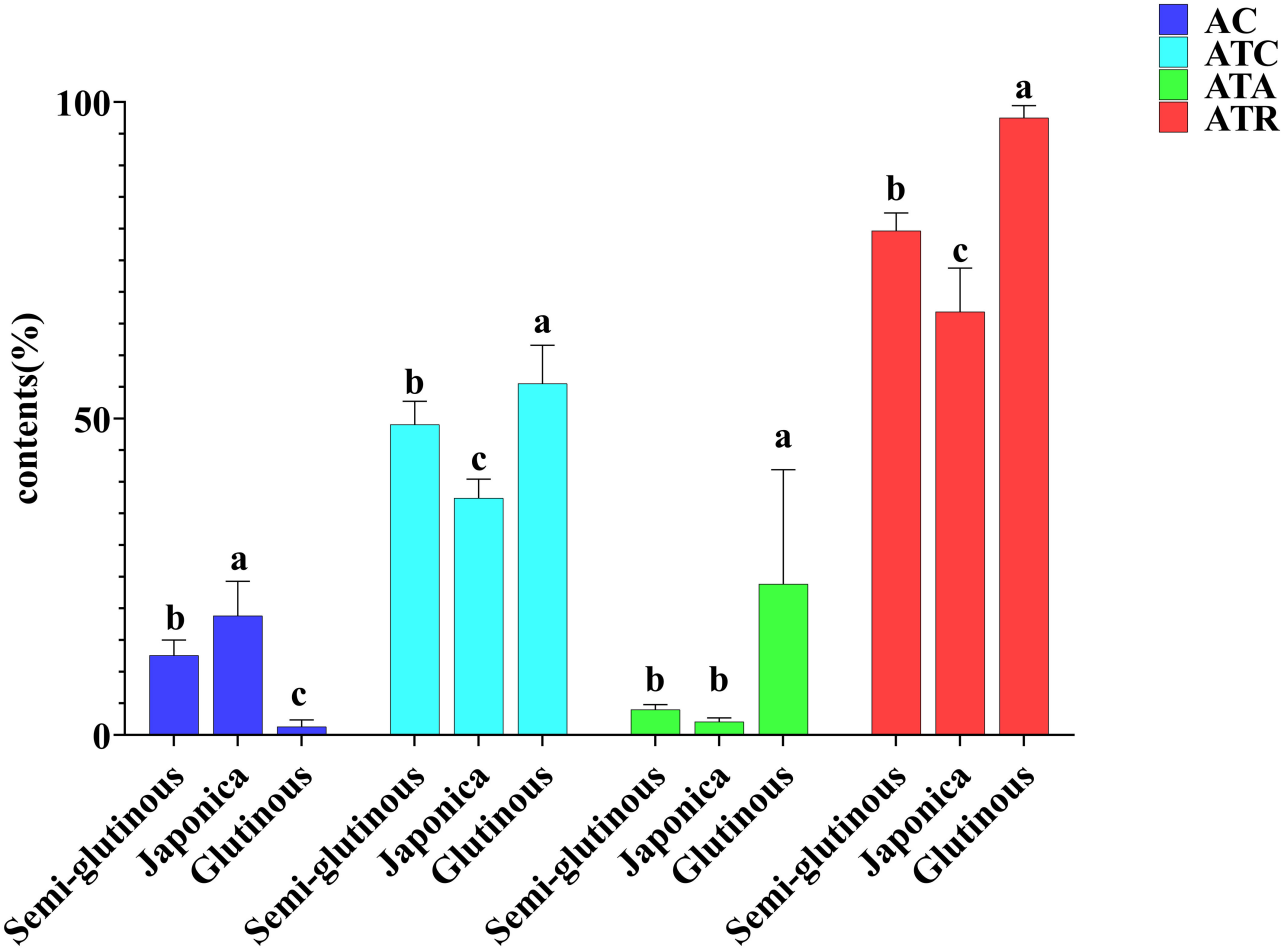
Comparison of starch fraction contents among different types of sorghum. Letters indicate significant differences at the 0.05 level of ANOVA.

Cluster analysis was performed on 19 lines using four indicators, AC, ATR, ATC and ATA. These 19 lines were classified into three groups: Cluster I containing 11 lines (2, 3, 5, 6, 7, 9, 10, 12, 13, 15, 17). Cluster II containing 4 lines (8, 16, 4, 14). Cluster III containing 4 lines (18, 19, 1, 11) (**Figure 3**). The amylose contents for the three clusters were as follows: Cluster I: 12.60%. Cluster II: 18.87% and Cluster III: 1.35%. The ATC were: Cluster I: 49.09%, Cluster II: 37.47% and Cluster III: 55.54%. ATA were: Cluster I: 4.10, Cluster II: 2.11 and Cluster III: 23.86. ATR were: Cluster I: 79.69, Cluster II: 66.90 and Cluster III: 97.51 (**Figure 4**). Based on these starch fractions: Cluster II had the highest amylose content and relatively low amylopectin content and branched-to-straight ratio, classifying it as a japonica sorghum. Cluster I had intermediate amylose and amylopectin contents with a relatively low branched-to-straight ratio, classifying it as a semi-glutinous sorghum. Cluster III had the lowest amylose content and the highest amylopectin content, indicating a significant glutinous characteristic, classifying it as a glutinous sorghum.

**Figure 5:**
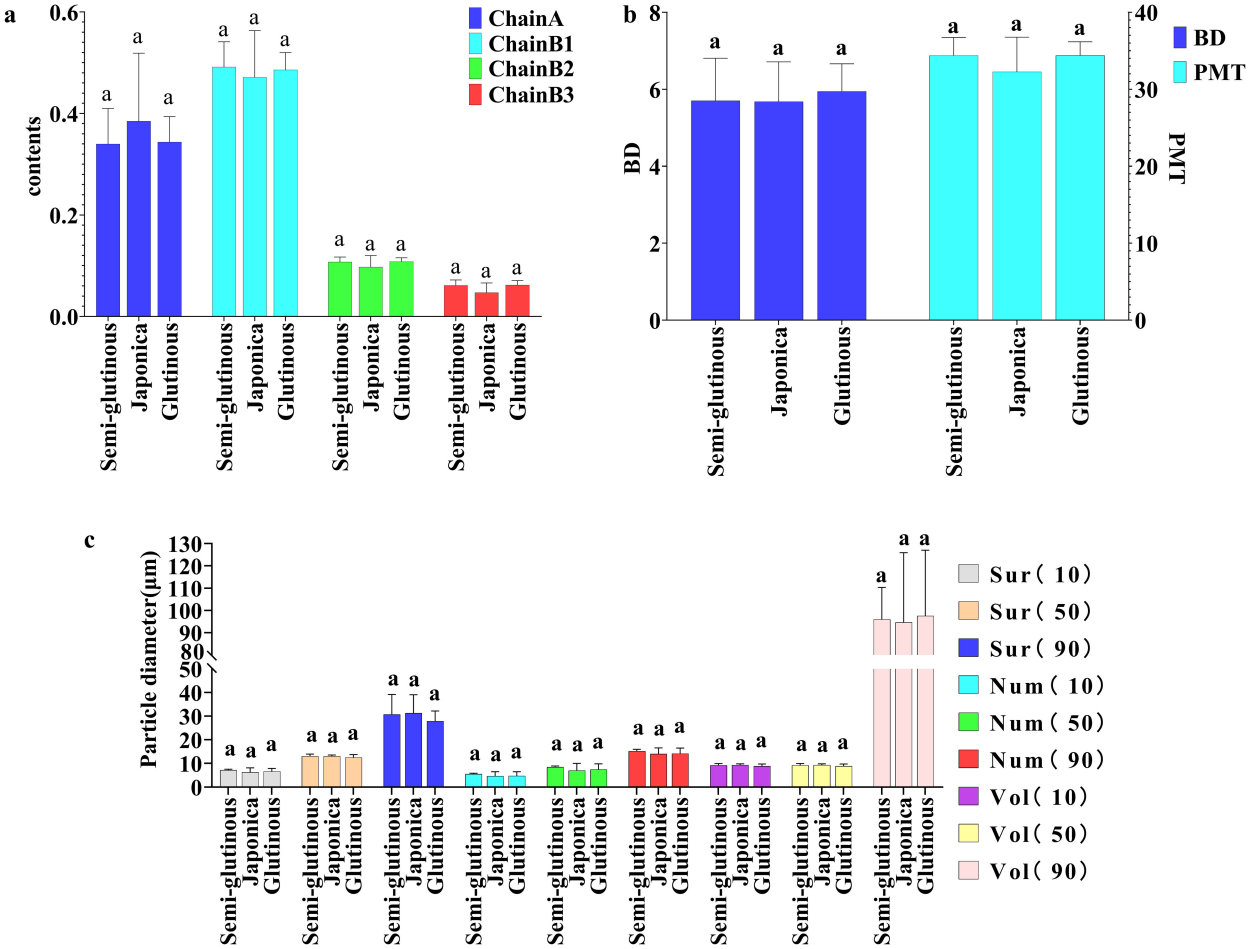
Comparison of structural properties among different types of sorghum. **(A)** Chain A, Chain B1, Chain B2 and Chain B3; **(B)** BD and PMT; **(C)** particle size distribution characteristics. Letters indicate significant differences at the 0.05 level of ANOVA.

Comparing the 15 structural properties indicators and 11 physicochemical property indicators among the three types of sorghums (glutinous sorghum, Japonica sorghum and semi-glutinous sorghum), the analysis revealed the following findings: Structural indicators: there were no significant differences among the structural indicators of glutinous sorghum, Japonica sorghum, and semi-glutinous sorghum (**Figure 5**).

**Figure 6:**
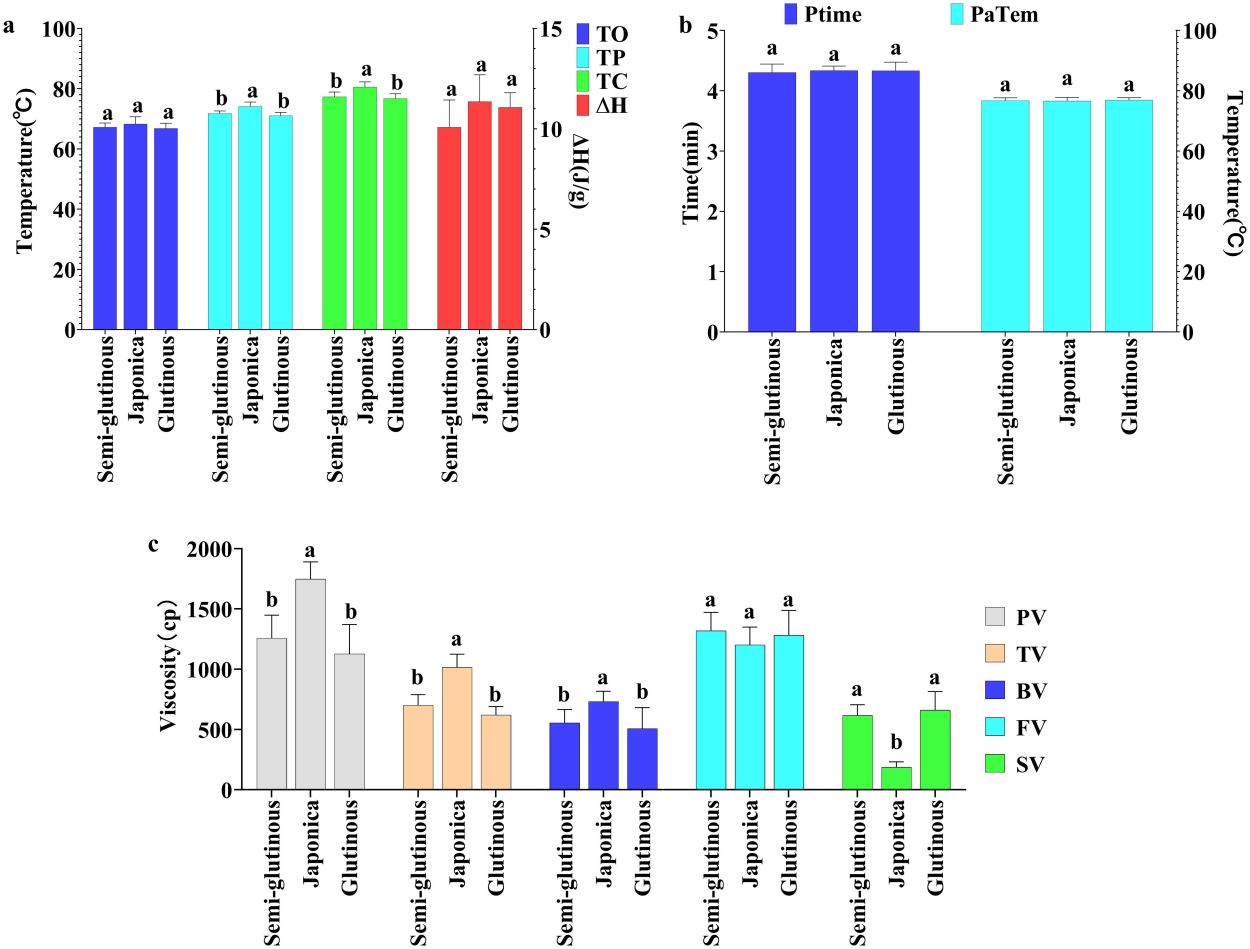
Comparison of physicochemical properties among different types of sorghum. **(A)** Thermodynamic properties; **(B)** Ptime and PaTem of pasting properties; **(C)** Viscosity indicators of pasting properties. Letters indicate significant differences at the 0.05 level of ANOVA. Letters indicate significant differences at the 0.05 level of ANOVA.

Physicochemical indicators: five physicochemical indicators (TP, TC, PV, TV, and BV) of Japonica glutinous sorghum, were significantly higher than those of glutinous and semi-glutinous sorghum, with no significant difference between glutinous and semi-glutinous sorghum. The setback viscosity (SV) of Japonica sorghum was significantly lower than that of glutinous sorghum and semi-glutinous sorghum, again with no significant difference between the latter two. There were no significant differences among the three types of sorghum in the five indicators: TO, ΔH, FV, PT, and PaTem (**Figure 6**). The results indicate that Japonica sorghum exhibits superior thermal stability and viscoelastic properties, making its processed products less likely to harden or lose texture during storage, which is a significant advantage for food products requiring long-term storage. Indicators that did not show significant differences may not be key factors in distinguishing between different types of sorghum or might not have a significant impact on sorghum applications.

## 4 Discussion

The competition between amylose and amylopectin during sorghum starch synthesis and the preference of different genotypes for the synthesis of these two starch types are largely influenced by genetic factors. These genetic factors include genes controlling enzymes related to starch synthesis, such as starch synthase (GBSS), starch branching enzyme (SBE), and starch debranching enzyme (DBE), etc. GBSS is the key enzyme for amylose synthesis, and its activity directly determines the content of amylose. Mutations in the GBSS gene or differences in its expression level lead to variations in amylose content. Sorghum varieties with different genotypes may have different GBSS enzyme activities and thus show a preference for amylose synthesis. SBE is a key enzyme in amylopectin synthesis and is responsible for the formation of branches in the starch chain. SBE activity, expression level, and genotype also affect amylopectin content and chain-length distribution. DBE acts as a modifying enzyme in the synthesis of starch and maintains the stability of the starch structure through the removal of unwanted branches. DBE plays a modifying role in starch synthesis by removing unwanted branches and maintaining the stability of the starch structure. The activity of DBE also affects the chain length distribution and properties of branched starch. Sorghum starch synthesis is a complex biochemical process involving multiple metabolic pathways. These metabolic pathways include glycolysis, pentose phosphate pathway, gluconeogenesis, and starch synthesis and degradation pathways. In the glycolysis and pentose phosphate pathways, glucose is converted to intermediates such as gluconic acid phosphate, fructose-6-phosphate, and glucose-6-phosphate, which are further converted to ADP-glucose, which serves as a substrate for starch synthesis. The activity of these metabolic pathways is regulated by a variety of enzymes, including glucose phosphate isomerase, fructose diphosphatase, and glucose-6-phosphatase. Changes in the activity of these enzymes also affect the rate and type of starch synthesis. During sorghum starch synthesis, there is competition between amylose and amylopectin. For example, both amylose and amylopectin synthesis depend on ADP-glucose as a substrate. Therefore, when the supply of ADP-glucose is limited, the synthesis of amylose and amylopectin compete with each other for substrate resources. Enzymes such as GBSS and SBE may have a competitive relationship in catalyzing the synthesis of amylose amylopectin. Differences in the activities, expression levels and genotypes of these enzymes affect the rate and ratio of amylose and amylopectin synthesis. Inside the starch granule, the molecular chains of amylose and amylopectin are intertwined to form a complex network structure. This structure results in spatial competition between amylose and amylopectin in the synthesis process.

Resistant starch is often associated with straight chain starches in other crops like wheat and rice, as its crystalline form offers resistance to enzymatic degradation (Cervini et al., 2021; Shen et al., 2022; Tiozon et al., 2023; Luo et al., 2023; Alsamadany et al., 2022; Irshad et al., 2022). However, in this study, no significant correlation was found between resistant starch content and any of the starch fractions. This lack of correlation may be attributed to the porous structure of sorghum starch, which could hinder the penetration of α-amylase (Cervini et al., 2021; Tiozon et al., 2023). The coefficient of variation for resistant starch content among 19 lines in this study was 130.49%, which not only indicated that there was high variability among lines, but also might be related to genetic polymorphisms related to resistant starch metabolizing enzymes. The process of resistant starch synthesis and catabolism involves the participation of many enzymes, and the activity, structure and expression levels of the enzymes related to starch synthesis and catabolism among different lines may be affected by genetic factors. In order to more accurately understand the degree and mechanism of the influence of these factors, more in-depth studies and experimental validation are needed in the future.

Short-chain starch molecules are smaller and easier to be dispersed and dissolved in water, so the pasting temperature is relatively low, and it is easier to form a single-molecule state during the pasting process, which is surrounded by water and melts to form a viscous paste solution. The long-chain starch has larger molecules and more compact structure, which needs higher temperature to make it disperse and dissolve, the pasting temperature is relatively high, and the viscosity of the solution formed after pasting is relatively low. When a certain balance is reached between short-chain and long-chain starch, the pasting temperature of starch will be moderate, neither too high nor too low, which is conducive to the processing and use of starch. Research has shown a significant negative correlation between the B3 chain content in rice and the starch breakdown value (Cai et al., 2014; Li et al., 2023; Tang et al., 2022). The size of starch granules usually exists in specific shapes, sizes and number ratios, and its particle size distribution is a complex parameter involving the range of granule sizes, distribution patterns, and so on. There are differences in the particle size distribution of different types of starch. For example, barley starch shows a wide particle size distribution, large granule starch is discoid, medium granule starch is oblate or ellipsoidal, and small granule starch is spherical or polygonal. In contrast, sweet potato starch can be classified into different levels such as small (6.67 μm), medium (11.54 μm) and large granular starches (16.96 μm) according to the median particle size (D50). Large granular starches usually have higher peak, disintegration and final viscosities, whereas small granular starches may have faster dissolution rates and higher fast-digesting starch content. Relative crystallinity increases with particle size, which may affect the enzymatic rate and chemical reaction activity of starch. Similarly, this study found a significant negative correlation between the B3 chain content in sorghum seed starch and its breakdown value, which measures the breakdown and fluidity of starch during heating. A higher breakdown value indicates greater starch breakdown and mobility during heating (Oladele et al., 2019; Zhu and Liu, 2020). In sorghum, significant correlations were observed between the average degree of polymerization, A chain content, B1 chain content, B2 chain content, and the TO.

Thermodynamic properties relate to starch’s stability and response to changing thermodynamic conditions, while pasting properties pertain to its physical and chemical changes during the heating process (Wasserman et al., 2023). Regarding thermodynamic properties, this study found that the peak and termination temperatures were primarily influenced by starch fractions, while the TO and pasting entropy were more affected by the structural properties of the starch. These findings align with previous studies on wheat (Cao et al., 2019; Zhang et al., 2013). The ratio and distribution of amylose and amylopectin directly impact the thermodynamic characteristics of starch (Wasserman et al., 2023). Generally, starches with higher amylose content have lower peak and termination temperatures, whereas those with higher amylopectin content have higher peak and termination temperatures (Oladele et al., 2019; Wasserman et al., 2023; Zhang et al., 2013). The size, shape, and internal structure of sorghum starch granules influence their onset pasting temperature. Larger granules have a relatively smaller surface area, requiring higher temperatures to allow water molecules to fully penetrate the interior and initiate pasting (Trappey et al., 2015). Intermolecular forces such as hydrogen bonding and van der Waals forces, also affect the onset pasting temperature of sorghum starch. These forces help maintain the stable structure of the starch granules prior to pasting, and a certain amount of energy is needed to overcome them (Cabrera-Ramírez et al., 2021; Li et al., 2024). In this study, 38.1% of the pasting characteristic indices showed significant or highly significant correlations with starch components, while only 9.5% had such correlations with structural characteristics. This suggests that the pasting characteristics of sorghum are primarily influenced by its starch components. Starch, being the main carbohydrate source in sorghum, directly affects the pasting characteristics through its content and type (Punia et al., 2020; Chen et al., 2022; Oladele et al., 2019; Chen et al., 2022). For instance, the ratio of amylose to amylopectin, starch granule size, and degree of crystallinity significantly impact properties like water absorption and swelling, pasting temperature, and pasting viscosity. Although structural properties do influence the pasting characteristics of sorghum, their effects are typically indirect, affecting the release of starch granules and water penetration (Punia et al., 2020; Wang et al., 2013). Consequently, statistically significant correlations between structural properties and pasting characteristics are less common compared to the direct effects of starch components (Huang and Lai, 2014).

Comparing the structural and physicochemical characteristics of three types of sorghum (glutinous sorghum, japonica sorghum, and semi-glutinous type sorghum) revealed both significant and non-significant differences. First, the non-significant differences in structural characteristics may be due to the genetically similar genomic structures of the three sorghum types, resulting in no significant structural differences. Additionally, similar environmental factors could contribute to their structural similarities Secondly, Japonica sorghum exhibited significant advantages in physicochemical properties. This is reflected in increased indicators such as TP, TC, and PV. Additionally, japonica sorghum usually has a higher content of straight-chain starch, which has a more linear molecular structure and is relatively less likely to form a hydrogen-bonding network and therefore may exhibit higher thermal stability and a wider range of pasting temperatures during heat treatment. This property is especially important in food processing, where breads or pastries made with Japonica sorghum starch are better able to retain their shape and texture during baking and are less likely to collapse or become overly fluffy in the baking industry. In contrast, some other starches such as maize starch or potato starch may be more susceptible to pasting at high temperatures, resulting in poor texture. The starch granules of Japonica sorghum have a smooth surface and a looser structure, and this structural property may make it easier to maintain stability during the heating process, thus affecting the changes in TP, TC and PV. In food processing, viscoelasticity is critical to the taste and texture of food. In noodle or pasta making, Japonica sorghum starch provides enough viscosity and elasticity to make noodles stronger and less likely to break, and pasta softer and chewier. This property makes Japonica sorghum ideal for making high-quality pasta (Adzqia et al., 2023; Alqah et al., 2020). The genetic characteristics of semi-glutinous sorghums fall between those of Japonica and glutinous sorghums, showing similar physicochemical properties to glutinous sorghum. These types also likely have similar environmental adaptations, contributing to the consistency in their physicochemical properties (Alqah et al., 2020). Indicators such as TP, ΔH, FV, Ptime, and PaTem may be influenced by a combination of factors including starch type, amylase activity, and moisture content. The three sorghum types may exhibit similar characteristics in these areas, resulting in no significant differences.

The main nutrients in sorghum grain contain not only starch but also proteins and lipids. There is a tight binding relationship between starch and protein in sorghum grain. Starch granules are usually embedded in a protein matrix, a structure that makes it difficult to completely separate starch granules during extraction. The structure and morphology of starch granules may also be affected by proteins. For example, the presence of proteins may alter the swelling behavior and pasting properties of starch granules. Also, the pasting state of starch may affect the solubility and conformational stability of proteins, which may further affect their functional properties. Proteins not only affect the structure of starch, but also have an impact on starch properties, due to the presence of interaction forces between proteins and starch may affect the pasting temperature, pasting speed and degree of pasting of starch. When the protein content is high, the pasting temperature of starch may increase, and the pasting speed may decrease. In addition, due to the wrapping and covering effect of protein on the starch granules, it makes it difficult for enzymes to reach the starch chains inside the starch granules, thus reducing the digestibility of the starch. It also results in lower amino acid utilization, thus affecting the overall nutritional value of sorghum grain. Lipids, usually in the form of free fatty acids or phospholipids, are mainly distributed on the surface of the starch granules or are trapped inside the starch granules. Forming straight-chain starch-lipid complexes with starch granules, their presence reduces enzyme-substrate contact, which affects the degree of starch solubility, pasting characteristics and enzyme digestibility, and they also impede the binding of starch granules to water molecules, which reduces the rate of pasting and viscosity. These interactions affect their food processing properties. For example, they affect the viscosity and ductility, digestibility and utilization of baked goods, while the presence of lipids may affect the fatty acid composition and energy density of foods.

## 5 Conclusion

In this study, 19 sorghum lines were used as research materials, leading to several key conclusions based on correlation analyses of key traits. By examining starch fractions, chain length distribution properties, particle size distribution properties, thermodynamic properties, and pasting properties, the following insights were gained:

Correlation between starch components and amylose structure: while the correlation between starch components and amylose structure is small, it significantly affects starch properties. The components and structure of starch together determine its thermodynamic properties and pasting characteristics (**Supplementary Material**).

Impact of starch structure on properties: amylose (straight chain starch) tends to reduce the viscosity and stability of starch pastes. In contrast, amylopectin (branched chain starch) enhances these properties.

Thermodynamic properties and pasting characteristics: the thermodynamic properties of starch directly influence its pasting characteristics. Understanding these relationships provides a theoretical basis for controlling starch behavior in food processing.

## Data Availability

The original contributions presented in the study are included in the article/**Supplementary Material**. Further inquiries can be directed to the corresponding authors.

## Supplementary material

Supplementary Figure 1 Characterization of chain length distribution across different sorghum lines. **(A)** Peak area; **(B)** chain length distribution; **(C)** average degree of aggregation; **(D)** degree of branching.

Supplementary Figure 2 Characterization of particle size distribution for different sorghum lines. **(A)** Specific surface area distribution; **(B)** Quantity distribution; **(C)** Volume distribution.

Supplementary Figure 3 Thermodynamic properties of different sorghum lines. **(A)** onset, peak and termination temperatures; **(B)** entropy of pasting.

Supplementary Figure 4 Brabender viscosity profiles for different sorghum lines.
