# Supplementary figures and images for: The correlation of starch composition, physicochemical and structural properties of different sorghum grains
Source: Front Plant Sci. 2025 Feb 25;16:1515022. doi: 10.3389/fpls.2025.1515022 (PMC11894258; doi:10.3389/fpls.2025.1515022)

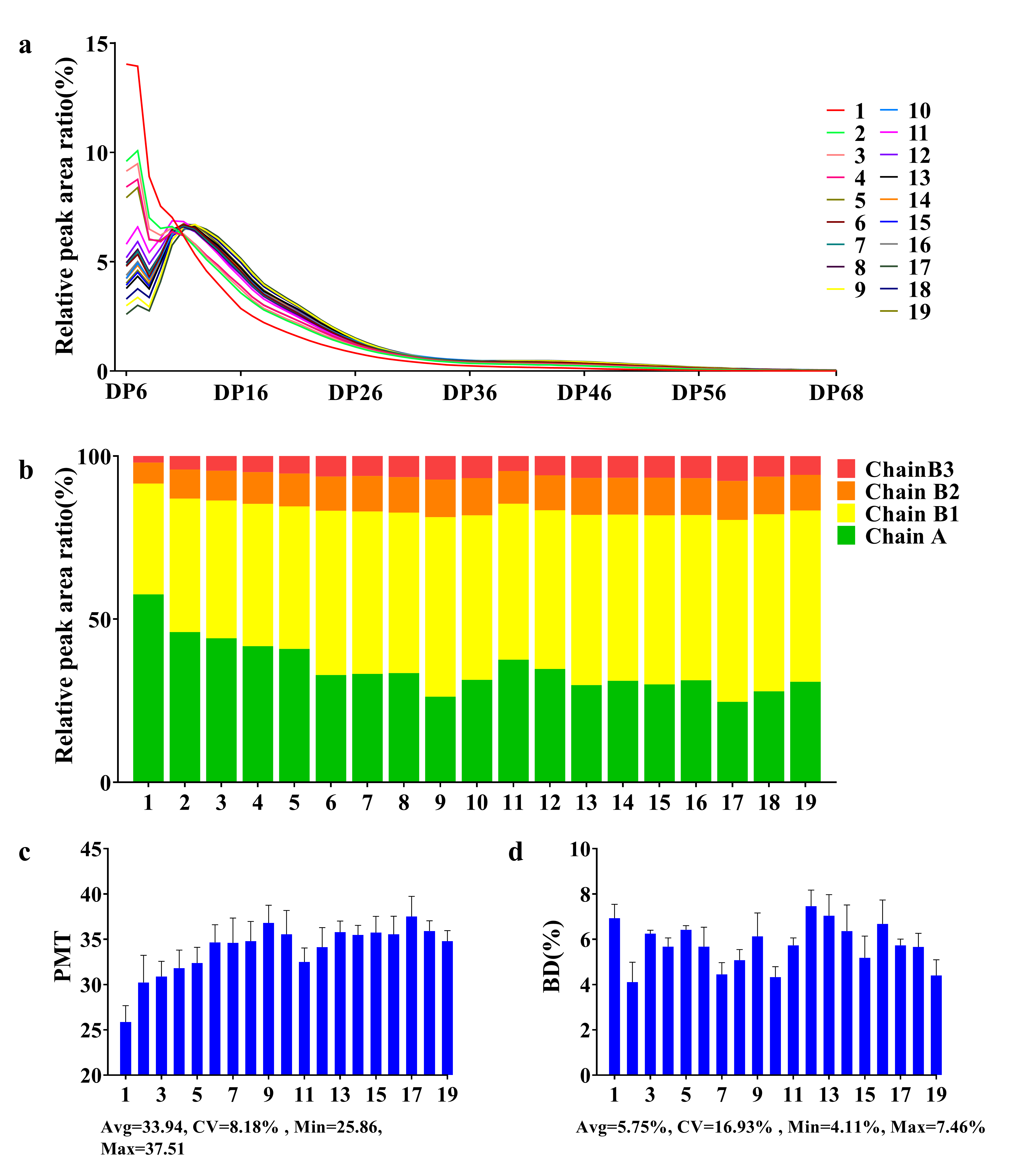

Supplement: Supplementary Figure 1 — Characterization of chain length distribution across different sorghum lines. (A) Peak area; (B) chain length distribution; (C) average degree of aggregation; (D) degree of branching. [file Image1.tif]

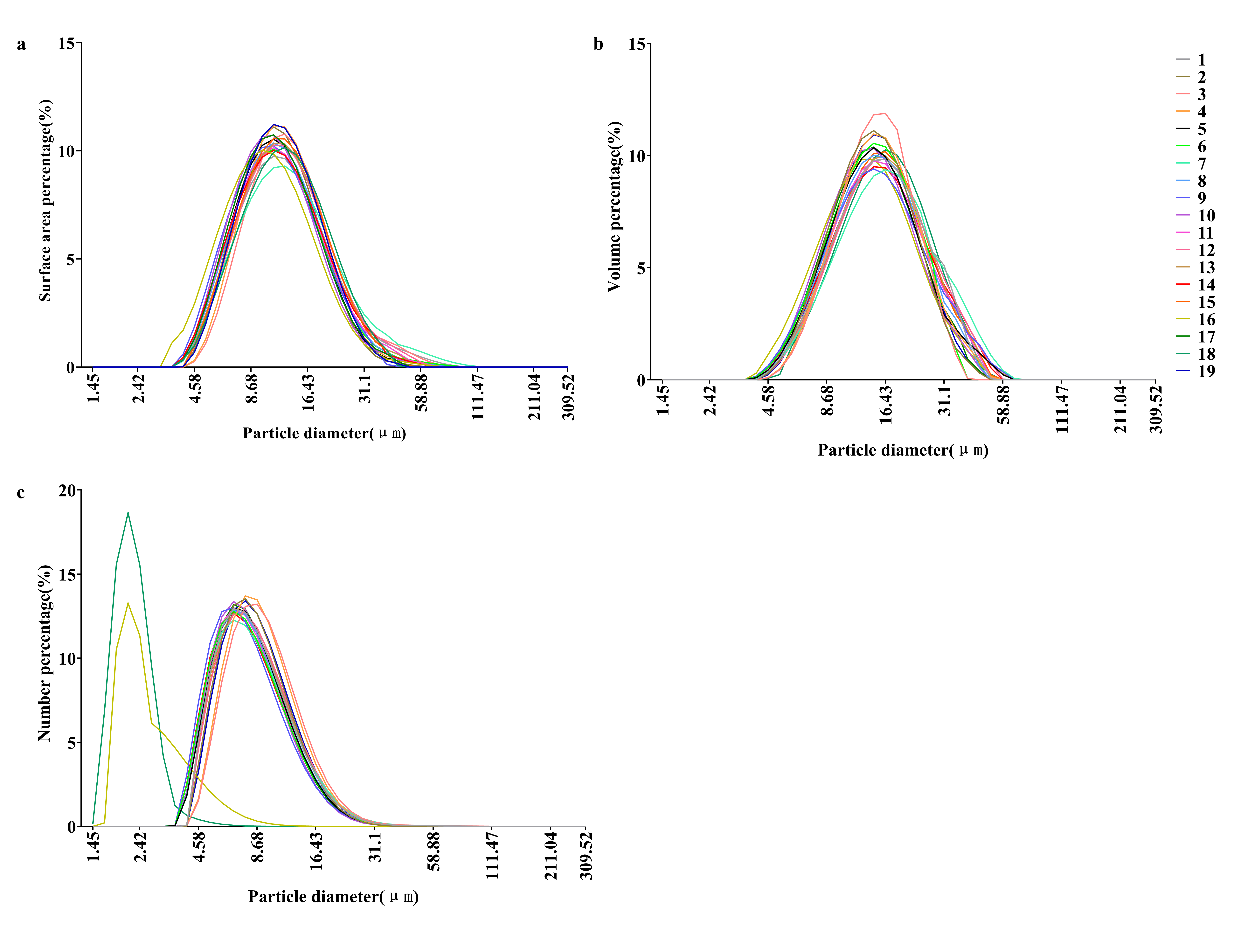

Supplement: Supplementary Figure 2 — Characterization of particle size distribution for different sorghum lines. (A) Specific surface area distribution; (B) Quantity distribution; (C) Volume distribution. [file Image2.tif]

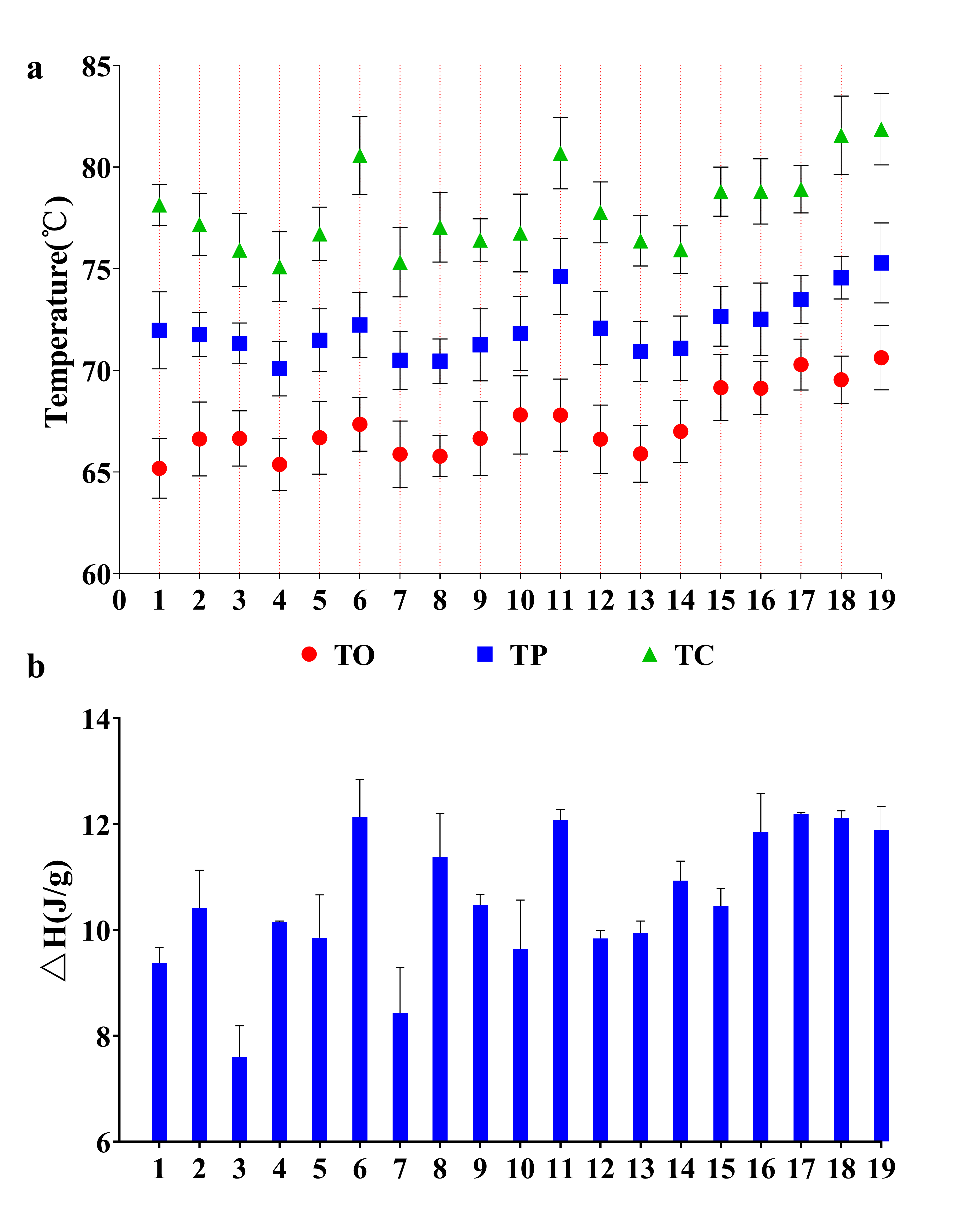

Supplement: Supplementary Figure 3 — Thermodynamic properties of different sorghum lines. (A) onset, peak and termination temperatures; (B) entropy of pasting. [file Image3.tif]

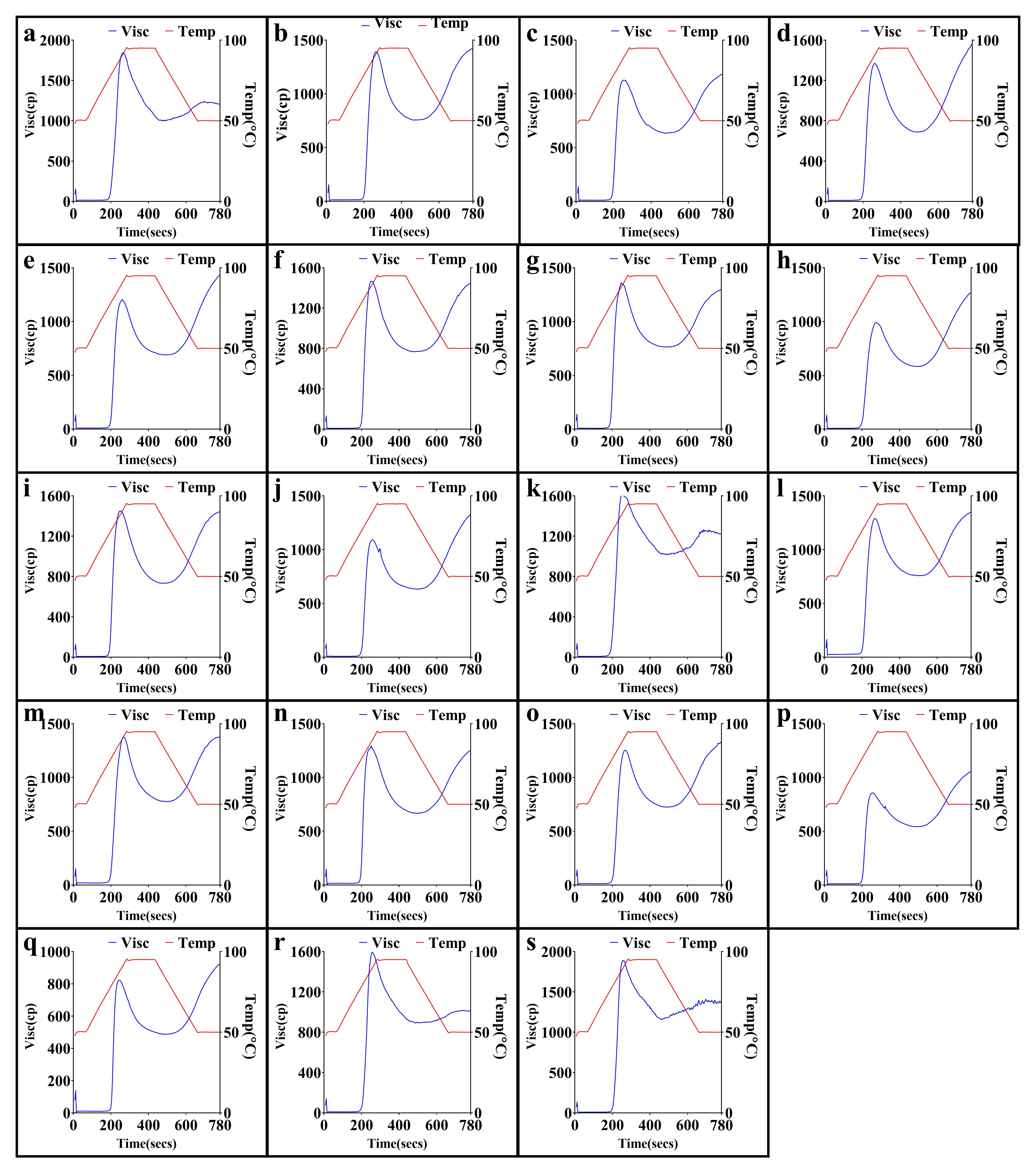

Supplement: Supplementary Figure 4 — Brabender viscosity profiles for different sorghum lines. [file Image4.tif]
