# Supplementary material for: The correlation of starch composition, physicochemical and structural properties of different sorghum grains
Source: Front Plant Sci. 2025 Feb 25;16:1515022. doi: 10.3389/fpls.2025.1515022 (PMC11894258; doi:10.3389/fpls.2025.1515022)
Supplement: Supplementary file 5 [file Table1.docx]

Supplemental Table1. Correlation analysis of starch traits in sorghum grain

| Items | SC | AC | RSC | ATC | ATR | ATA | BD | PMT | Chain A | Chain B1 | Chain B2 | Chain B3 | Sur(10) | Sur(50) | Sur(90) | Num(10) | Num(50) | Num(90) | Vol(10) | Vol(50) | Vol(90) | PV | TV | BV | FV | SV | Ptime | PaTem | TO | TP | TC | ΔH |
| --- | --- | --- | --- | --- | --- | --- | --- | --- | --- | --- | --- | --- | --- | --- | --- | --- | --- | --- | --- | --- | --- | --- | --- | --- | --- | --- | --- | --- | --- | --- | --- | --- |
| SC | 1 |  |  |  |  |  |  |  |  |  |  |  |  |  |  |  |  |  |  |  |  |  |  |  |  |  |  |  |  |  |  |  |
| AC | 0.307 | 1 |  |  |  |  |  |  |  |  |  |  |  |  |  |  |  |  |  |  |  |  |  |  |  |  |  |  |  |  |  |  |
| RSC | 0.184 | 0.114 | 1 |  |  |  |  |  |  |  |  |  |  |  |  |  |  |  |  |  |  |  |  |  |  |  |  |  |  |  |  |  |
| ATC | 0.496* | -0.675** | 0.038 | 1 |  |  |  |  |  |  |  |  |  |  |  |  |  |  |  |  |  |  |  |  |  |  |  |  |  |  |  |  |
| ATR | -0.145 | -0.984** | -0.095 | 0.785** | 1 |  |  |  |  |  |  |  |  |  |  |  |  |  |  |  |  |  |  |  |  |  |  |  |  |  |  |  |
| ATA | -0.375 | -0.638** | -0.257 | 0.291 | 0.639** | 1 |  |  |  |  |  |  |  |  |  |  |  |  |  |  |  |  |  |  |  |  |  |  |  |  |  |  |
| BD | 0.071 | 0.156 | -0.166 | -0.087 | -0.153 | -0.145 | 1 |  |  |  |  |  |  |  |  |  |  |  |  |  |  |  |  |  |  |  |  |  |  |  |  |  |
| PMT | 0.466* | 0.420 | 0.161 | -0.022 | -0.351 | -0.077 | -0.119 | 1 |  |  |  |  |  |  |  |  |  |  |  |  |  |  |  |  |  |  |  |  |  |  |  |  |
| Chain A | -0.45 | -0.335 | -0.142 | -0.043 | 0.265 | 0.006 | 0.129 | -0.994** | 1 |  |  |  |  |  |  |  |  |  |  |  |  |  |  |  |  |  |  |  |  |  |  |  |
| Chain B1 | 0.435 | 0.256 | 0.126 | 0.104 | -0.184 | 0.058 | -0.132 | 0.978** | -0.995** | 1 |  |  |  |  |  |  |  |  |  |  |  |  |  |  |  |  |  |  |  |  |  |  |
| Chain B2 | 0.439 | 0.418 | 0.139 | -0.041 | -0.358 | -0.094 | -0.151 | 0.989** | -0.978** | 0.955** | 1 |  |  |  |  |  |  |  |  |  |  |  |  |  |  |  |  |  |  |  |  |  |
| Chain B3 | 0.485* | 0.548* | 0.198 | -0.124 | -0.480* | -0.179 | -0.086 | 0.985** | -0.962** | 0.932** | 0.971** | 1 |  |  |  |  |  |  |  |  |  |  |  |  |  |  |  |  |  |  |  |  |
| Sur(10) | -0.018 | 0.078 | 0.207 | -0.085 | -0.093 | -0.466* | -0.068 | -0.357 | 0.380 | -0.396 | -0.353 | -0.311 | 1 |  |  |  |  |  |  |  |  |  |  |  |  |  |  |  |  |  |  |  |
| Sur(50) | -0.173 | -0.290 | 0.207 | 0.130 | 0.270 | 0.041 | 0.079 | -0.413 | 0.396 | -0.378 | -0.407 | -0.433 | 0.586** | 1 |  |  |  |  |  |  |  |  |  |  |  |  |  |  |  |  |  |  |
| Sur(90) | -0.035 | -0.187 | 0.338 | 0.144 | 0.189 | -0.047 | 0.272 | -0.483* | 0.474* | -0.458 | -0.503* | -0.475* | 0.390 | 0.788** | 1 |  |  |  |  |  |  |  |  |  |  |  |  |  |  |  |  |  |
| Num(10) | -0.001 | 0.002 | 0.201 | -0.002 | -0.007 | -0.367 | -0.144 | -0.328 | 0.341 | -0.350 | -0.328 | -0.297 | 0.971** | 0.603** | 0.324 | 1 |  |  |  |  |  |  |  |  |  |  |  |  |  |  |  |  |
| Num(50) | 0.016 | 0.058 | 0.205 | -0.041 | -0.064 | -0.434 | -0.115 | -0.322 | 0.341 | -0.355 | -0.322 | -0.282 | 0.990** | 0.575* | 0.332 | 0.993** | 1 |  |  |  |  |  |  |  |  |  |  |  |  |  |  |  |
| Num(90) | -0.049 | -0.023 | 0.201 | -0.017 | 0.010 | -0.347 | -0.070 | -0.359 | 0.371 | -0.377 | -0.355 | -0.333 | 0.971** | 0.734** | 0.456* | 0.973** | 0.975** | 1 |  |  |  |  |  |  |  |  |  |  |  |  |  |  |
| Vol(10) | -0.187 | -0.297 | 0.223 | 0.126 | 0.275 | 0.032 | 0.111 | -0.512* | 0.498* | -0.480* | -0.510* | -0.521* | 0.582** | 0.982** | 0.863** | 0.584** | 0.561* | 0.710** | 1 |  |  |  |  |  |  |  |  |  |  |  |  |  |
| Vol(50) | -0.071 | -0.209 | 0.303 | 0.136 | 0.206 | -0.029 | 0.279 | -0.526* | 0.514* | -0.493* | -0.552* | -0.520* | 0.389 | 0.807** | 0.979** | 0.335 | 0.340 | 0.472* | 0.879** | 1 |  |  |  |  |  |  |  |  |  |  |  |  |
| Vol(90) | -0.152 | -0.021 | 0.157 | -0.099 | -0.006 | -0.249 | 0.107 | -0.569* | 0.585** | -0.588** | -0.592** | -0.519* | 0.562* | 0.479* | 0.563* | 0.516** | 0.539* | 0.560* | 0.575* | 0.661** | 1 |  |  |  |  |  |  |  |  |  |  |  |
| PV | -0.476* | -0.739** | -0.104 | 0.305 | 0.696 | 0.595** | -0.065 | -0.415 | 0.332 | -0.252 | -0.435 | -0.532* | 0.044 | 0.333 | 0.140 | 0.138 | 0.083 | 0.160 | 0.304 | 0.218 | 0.126 | 1 |  |  |  |  |  |  |  |  |  |  |
| TV | -0.384 | -0.813** | -0.188 | 0.444 | 0.789 | 0.637** | -0.103 | -0.356 | 0.267 | -0.185 | -0.360 | -0.491* | 0.009 | 0.269 | 0.093 | 0.085 | 0.040 | 0.113 | 0.240 | 0.161 | 0.098 | 0.955** | 1 |  |  |  |  |  |  |  |  |  |
| BV | -0.529* | -0.563* | 0.008 | 0.104 | 0.503 | 0.475* | -0.012 | -0.437 | 0.370 | -0.302 | -0.473* | -0.517* | 0.080 | 0.369 | 0.180 | 0.185 | 0.124 | 0.197 | 0.344 | 0.259 | 0.144 | 0.935** | 0.788** | 1 |  |  |  |  |  |  |  |  |
| FV | -0.409 | 0.036 | 0.286 | -0.351 | -0.128 | -0.257 | -0.146 | -0.197 | 0.218 | -0.238 | -0.169 | -0.170 | 0.539* | 0.214 | -0.121 | 0.572* | 0.564* | 0.538* | 0.166 | -0.071 | 0.289 | 0.317 | 0.194 | 0.425 | 1 |  |  |  |  |  |  |  |
| SV | -0.008 | 0.680** | 0.372 | -0.627** | -0.731** | -0.709** | -0.031 | 0.133 | -0.046 | -0.036 | 0.157 | 0.262 | 0.409 | -0.050 | -0.168 | 0.375 | 0.405 | 0.325 | -0.064 | -0.184 | 0.145 | -0.520* | -0.651** | -0.303 | 0.618** | 1 |  |  |  |  |  |  |
| Ptime | -0.284 | -0.206 | -0.364 | -0.033 | 0.145 | -0.075 | 0.185 | -0.337 | 0.345 | -0.352 | -0.292 | -0.343 | 0.183 | 0.176 | -0.033 | 0.182 | 0.181 | 0.221 | 0.185 | 0.075 | 0.450 | 0.153 | 0.203 | 0.076 | 0.336 | 0.098 | 1 |  |  |  |  |  |
| PaTem | 0.055 | 0.103 | -0.458* | -0.051 | -0.074 | 0.269 | -0.184 | 0.249 | -0.226 | 0.201 | 0.275 | 0.267 | -0.275 | -0.422 | -0.600** | -0.245 | -0.256 | -0.301 | -0.444 | -0.608** | -0.343 | -0.307 | -0.197 | -0.401 | -0.148 | 0.044 | 0.266 | 1 |  |  |  |  |
| TO | 0.327 | -0.135 | -0.278 | 0.377 | 0.221 | 0.422 | -0.255 | 0.530* | -0.571* | 0.599** | 0.531* | 0.452 | -0.451 | -0.476* | -0.441 | -0.424 | -0.433 | -0.476* | -0.516* | -0.523* | -0.628** | -0.076 | 0.094 | -0.268 | -0.521* | -0.478* | -0.352 | 0.481* | 1 |  |  |  |
| TP | 0.195 | -0.578** | -0.388 | 0.679** | 0.651** | 0.602** | -0.143 | 0.157 | -0.237 | 0.305 | 0.152 | 0.024 | -0.411 | -0.274 | -0.260 | -0.348 | -0.380 | -0.386 | -0.291 | -0.287 | -0.407 | 0.374 | 0.550* | 0.122 | -0.452 | -0.790** | -0.117 | 0.269 | 0.822** | 1 |  |  |
| TC | 0.133 | -0.527** | -0.463* | 0.585** | 0.587** | 0.599** | -0.114 | 0.148 | -0.223 | 0.291 | 0.120 | 0.032 | -0.474* | -0.321 | -0.329 | -0.414 | -0.441 | -0.442 | -0.335 | -0.313 | -0.304 | 0.396 | 0.538* | 0.180 | -0.362 | -0.711** | 0.021 | 0.351 | 0.728** | 0.919** | 1 |  |
| ΔH | 0.170 | -0.002 | -0.558** | 0.133 | 0.035 | 0.226 | -0.096 | 0.438 | -0.478* | 0.512* | 0.407 | 0.372 | -0.519* | -0.658** | -0.694** | -0.460* | -0.472* | -0.553* | -0.690** | -0.661** | -0.423 | 0.050 | 0.126 | -0.046 | -0.287 | -0.323 | -0.059 | 0.379 | 0.617** | 0.614** | 0.732** | 1 |

Note: "*" indicates p<0.05; "**" indicates p<0.01.
